# Relationship between cold pressor pain-sensitivity and sleep quality in opioid-dependent males on methadone treatment

**DOI:** 10.7717/peerj.839

**Published:** 2015-04-09

**Authors:** Zalina Zahari, Chee Siong Lee, Soo Choon Tan, Nasir Mohamad, Yeong Yeh Lee, Rusli Ismail

**Affiliations:** 1 Department of Pharmacy, Hospital Universiti Sains Malaysia, Kubang Kerian, Kelantan, Malaysia; 2 Pharmacogenetics and Novel Therapeutics Cluster, Institute for Research in Molecular Medicine (INFORMM), Universiti Sains Malaysia, Kubang Kerian, Kelantan, Malaysia; 3 Department of Emergency Medicine, School of Medical Sciences, Kubang Kerian, Kelantan, Malaysia; 4 Faculty of Medicine & Health Sciences, Universiti Sultan Zainal Abidin, Kuala Terengganu, Terengganu, Malaysia; 5 School of Medical Sciences, Universiti Sains Malaysia, Kubang Kerian, Kelantan, Malaysia; 6 Centre of Excellence for Research in AIDS (CERiA), University of Malaya, Kuala Lumpur, Malaysia

**Keywords:** Methadone maintenance therapy, Pain, Sleep quality, Opioid-dependence, Pain-tolerance, Pain-sensitivity

## Abstract

**Aim.** Poor sleep quality due to pain has been reported among opioid-dependent male patients on methadone maintenance therapy (MMT) but objective pain data are lacking. This study aimed to investigate the rate of pain-sensitivity using cold pressor test (CPT) and the relationship between pain-sensitivity and sleep quality in this population.

**Methods.** A total of 168 male participants were included into the study. Objective pain-tolerance was evaluated at 0 h and at 24 h after the first CPT. Malay version of the Pittsburgh Sleep Quality Index (PSQI) and the subjective opiate withdrawal scale (SOWS) questionnaires were administered to evaluate the quality of sleep and withdrawal symptoms, respectively.

**Results.** The mean age of study participants was 37.22 (SD 6.20) years old. Mean daily methadone dose was 76.64 (SD 37.63) mg/day, mean global PSQI score was 5.47 (SD 2.74) and mean averaged SOWS score was 5.43 (SD 6.91). The averaged pain-tolerance time ranged from 7 to 300 s with a mean time of 32.16 (SE 2.72) s, slightly below the cut-off score of 37.53 s. More specifically, 78.6% (*n* = 132) of participants were identified as pain-sensitive (averaged pain-tolerance time ≤37.53 s), and 36 (21.4%) participants were pain-tolerant (averaged pain-tolerance time >37.53 s). The pain-sensitive group reported poorer sleep quality with mean (SD) PSQI of 5.78 (2.80) compared with the pain-tolerant group with mean (SD) PSQI of 4.31 (2.18) (*p* = 0.005). With analysis of covariance, pain-sensitive group was found to have higher global PSQI scores (adjusted mean 5.76, 95% CI 5.29; 6.22) than pain-tolerant participants (adjusted mean 4.42, 95% CI 3.52; 5.32) (*p* = 0.010).

**Conclusions.** Majority of opioid-dependent male patients on methadone treatment are pain-sensitive with CPT. Poor sleep quality is associated with cold pressor pain-sensitivity. Pain and sleep complaints in this male population should not be overlooked.

## Introduction

Pain is complex, subjective and has wide inter-individual variability (Fillingim, 2005; Coghill, 2010; Sato et al., 2013). Recent studies indicated that acute and chronic pain were common among a largely male patients on methadone maintenance therapy (MMT) (Peles, Schreiber & Adelson, 2006; Eyler, 2013). For example, a one-time self-report survey found that, out of 227 methadone-maintained patients from a single clinic setting in Baltimore, 137 patients (60%) had chronic pain (Dunn, Brooner & Clark, 2014). Inadequate pain management may contribute to failure in achieving overall treatment outcomes with methadone treatment (Hines et al., 2008; Eyler, 2013).

In recent years, attention has shifted to quality of sleep in patients on methadone treatment (Stein et al., 2004; Peles, Schreiber & Adelson, 2006; Peles, Schreiber & Adelson, 2009; Sharkey et al., 2009; Sharkey et al., 2010; Liao et al., 2011; Sharkey et al., 2011). For example, in a study that systematically studied the prevalence of sleep disorders among 135 heroin-dependent Chinese on MMT in Hunan Province, China the authors found that a majority of the patients (*n* = 134 subjects, 99.23%) had sleep disturbance with a total PSQI score of >5 (Liao et al., 2011). It is possible that the relationship between sleep disturbance and pain might be circular or reciprocal, with disturbed sleep contributing to enhanced pain-sensitivity, or disturbed sleep is caused by pain (Smith & Haythornthwaite, 2004; Edwards et al., 2008).

Although a number of studies have assessed the association between pain experience and sleep in patients on MMT (Stein et al., 2004; Peles, Schreiber & Adelson, 2006; Peles, Schreiber & Adelson, 2009), less is known about the relationship between pain-sensitivity and sleep quality. Existing studies only evaluated pain experience using self-reported pain questionnaires (Stein et al., 2004; Peles, Schreiber & Adelson, 2006; Peles, Schreiber & Adelson, 2009) but not with a quantitative test. The cold pressor test (CPT) is a standardized and naturalistic pain model, producing pain analogous to naturally occurring type of pain and effectively mimics chronic pain conditions such as dental and back pain (Chen et al., 1989). The reliability and validity of the cold pain model has been established with good validity and test–retest reliability (Chen et al., 1989; Ruscheweyh et al., 2010; Lewis et al., 2012).

The objectives of our study were to determine the rate of pain-sensitivity among male patients on methadone treatment and the relationship between pain-sensitivity and sleep quality. Objective pain testing using CPT rather than a pain questionnaire was used to measure pain-sensitivity in the current study.

## Methods

### Participants

The sample population consisted of opioid-dependent male patients who were undergoing methadone treatment between March and October 2013 at Hospital Universiti Sains Malaysia (USM) and other MMT clinics (Kota Bharu, Pasir Mas, Pasir Puteh and Bachok) in the state of Kelantan, Malaysia. Opioid dependency was defined according to the DSM IV criteria. American Psychiatric Association (1994). Study participants were included if they were (a) in the national MMT programme with a duration of participation of more than one month; (b) more than 18 years of age; (c) free of regular use of alcohol; (d) free of intoxication; (e) able to understand study protocols and to follow simple study instructions; and (f) willing to sign written informed consent. Exclusion criteria included the following: (a) presence of acute medical, surgical or psychiatric illnesses; (b) current intake of benzodiazepines, cannabinoids or barbiturates; (c) on regular anticonvulsants, neuroleptics or analgesics; (d) history of chronic or ongoing acute pain; (e) history of analgesics ingestion within 3 days before the CPT; and (f) the presence of severe cognitive impairment which might interfere with pain assessments and/or communication. Although no standard scales were used, psychiatric illnesses were carefully evaluated during interview of participants, and the information verified with their medical records and also from their doctors.

This study was approved by the Human Research Ethics Committee (HREC), Universiti Sains Malaysia (USM) in Kelantan, Malaysia (Reference number: USMKK/PPP/JEPeM (253.3 [14]) and the Medical Research & Ethics Committee (MREC) at the Ministry of Health (MOH), Malaysia (Reference number: NMRR-13-524-16614).

### Study procedure

All included study participants would be interviewed to obtain detailed demographic and clinical data including details related to their past drug history and recent drug use, and other treatment variables. All interviews were conducted by one interviewer (ZZ), with experience of five or more years of managing opioid-dependence. Subjects were asked to complete two validated Malay-language questionnaires i.e., the Pittsburgh Sleep Quality Index (PSQI-M) and subjective opiate withdrawal scale (SOWS-M). The SOWS-M was scored at 0 h (i.e., approximately 30 min before taking their morning dose of methadone), and at 24 h after the dose intake. Participants would be asked for any history of analgesics consumption within 72 h prior to CPT and for a history of any chronic and acute painful conditions. The CPT took place in a quiet, dedicated area at the Clinical Trial Unit (CTU), USM and was administered by a trained research assistant (SHH).

Details of study instruments are described below.

### Instruments

#### Cold pressor test (CPT)

The CPT method utilized in the current study was adapted based on previous reports from Chen et al. (1989) and Compton, Charuvastra & Ling (2001). The CPT apparatus consisted of a 48 quart cool box with two-thirds of crushed ice and one-third of tap water. A constant temperature of 0–2 °C was maintained by stirring the ice-water mixture and by adding ice intermittently. The temperature was monitored by a digital indoor-outdoor-thermometer (TFA Dostmann GmbH & Co. KG, Wertheim, Germany).

A standardized written instruction was read out to all participants before the first CPT. They were instructed to place their non-dominant hand and also forearm in the ice-water bath with their palm flat at the bottom of the box, so that ice-water mixture would cover their hand and approximately 10 cm of their forearm. The test was truncated at 300 s, since after this time point the numbness would set in and the pain diminished (Wolf & Hardy, 1941; Harris & Rollman, 1983; Compton, Charuvastra & Ling, 2001).

Pain-tolerance was defined as the most severe pain that a participant was willing to tolerate (i.e., the time elapsed when the participant withdrew his hand after immersion). Pain-tolerance to CPT was evaluated at 0 h and repeated at 24 h. Maximal time for pain-tolerance was 300 s. After withdrawal of the immersed hand, the participant was given a piece of dry towel to dry their hand.

#### Malay version of the Pittsburgh sleep quality index (PSQI-M)

The PSQI is a self-administered questionnaire to measure sleep quality and disturbances during the previous month and to discriminate between good and poor sleepers (Buysse et al., 1989). It is a validated questionnaire that has been translated into several languages including the Malay language (PSQI-M). Permission for use of PSQI-M was first obtained from the authors (Buysse et al., 1989). Information on the administration and scoring instructions are available from the website of the Sleep Medicine Institute, University of Pittsburgh (University of Pittsburgh Sleep Medicine Institute , 2013).

The 19 individual items in the questionnaire are used to generate seven component scores: subjective sleep quality (one item), sleep latency (two items), sleep duration (one item), habitual sleep efficiency (three items), sleep disturbances (nine items), use of sleep medications (one item), and daytime dysfunction (two items). Each of the seven component scores has a score range of 0–3 and the sum of seven component scores yielded one global score with a score range of 0–21, where a higher scores indicating a poorer sleep quality.

#### Subjective opiate withdrawal scale (SOWS-M)

The SOWS measures the severity of opiate withdrawal syndrome over a wide range of common signs and symptoms (Jain, Jain & Dhawan, 2011; Salehi et al., 2011; Chawla et al., 2013; Mustafa et al., 2013). A valid and reliable instrument (Handelsman et al., 1987), the SOWS is available in the Malay language (SOWS-M) (Mohamed Nazar, 2013) and the permission was acquired from authors prior to use in the current study.

The SOWS-M is a self-administered scale which contains 16 symptoms. Each symptom is rated on a 4-point Likert scale with a total score between 0 and 64. The SOWS-M was administered at two time points during the study; at 0 h i.e., approximately 30 min before taking their morning dose of methadone and at 24 h after the dose intake.

#### Urine drug test

Urine drug test screens for morphine, tetrahydrocannabinol, amphetamines and benzodiazepines (F.A.C.T.S ™ 4 in 1 Combo Dipcard Rapid Test (MOR/THC/AMP/BZO), Scientifacts Sdn. Bhd., Malaysia) and the test was performed in each participant twice in one week prior to the first CPT. Participants should have two consecutive negative urine tests before study inclusion.

### Data and statistical analysis

Mean and standard deviation (SD) were determined for continuous variables unless otherwise stated. By using the averaged pain-tolerance time (i.e., the average of two measurements taken at 0 h and at 24 h) cut-off of 37.53 s (taken as the mean plus two standard error, i.e., upper bound of 95% confidence interval for mean), participants were categorized into “pain-sensitive” (≤37.53 s) and “pain-tolerant” (>37.53 s) groups (Neziri et al., 2011).

A *t*-test was used to compare differences between averaged SOWS scores and mean global PSQI scores with characteristics of pain-sensitive and pain-tolerant groups including age, age of first illicit drug use, age of first opioid abuse, duration of opioid exposure, duration of opioid addiction, duration of illicit drug use prior to joining MMT, duration in MMT and methadone dose. Analysis of co-variance (ANCOVA) was performed to compare differences between the mean global PSQI scores with pain-sensitive and pain-tolerant groups by taking the duration of opioid addiction as the covariate. All analyses were performed using SPSS version 22 (SPSS Inc., Chicago, USA). The limit of significance was set to 0.05.

## Results

### Characteristics of study participants

Of 169 eligible participants, 168 (99.4%) agreed to participate in this study. One participant did not take part because a lack of interest. Almost all participants were of ethnic Malays (98.8%) and only two were Chinese. The mean age of study participants was 37.22 (SD = 6.20, range: 25–55) years old. The mean duration for participation in the MMT program was 2.92 (SD = 2.09, range: 0.33–9.00) years. The mean daily methadone dose was 76.64 (SD = 37.63, range: 20–360) mg/day. The mean averaged SOWS score was 5.43 (SD = 6.91, range: 0–48).

The demographic and clinical characteristics of study participants are shown in Table 1. The two groups were well matched with respect to age, age of first illicit drug use, age of first opioid abuse, duration of opioid exposure, duration of illicit drug use prior to joining MMT, duration in MMT, methadone dose, averaged SOWS score and monthly income. The averaged pain-tolerance time ranged from 7 to 300 s with a mean time of 32.16 (SD = 35.25) s, only slightly below the cut-off score of 37.53 s. Most participants (78.6% or 132 participants) were identified as pain-sensitive and only a minority (21.4% or 36 participants) were pain-tolerant. There was no significant relationship between pain-sensitivity and methadone dose (*p* = 0.81) (Table 1). However, duration of opioid addiction was significantly different between pain-sensitive and pain-tolerant groups (*p* = 0.044).

**Table 1:** Demographic and participants’ characteristics.

	Total (*N* = 168)		Pain-tolerant (*N* = 36)		Pain-sensitive (*N* = 132)				
Variable	Mean	SD	Mean	SD	Mean	SD	Mean difference (95% CI)	*t*-statistic (df)	*p*-value^a^
Age (years)	37.22	6.20	36.11	5.68	37.52	6.32	−1.41 (−3.71, 0.89)	−1.21 (166)	0.227
Age of first illicit drug use (years)	19.24	4.17	20.20	5.05	18.98	3.88	1.22 (−0.34, 2.78)	1.55 (163)	0.124
Age of first opioid abuse (years)	22.24	4.86	22.80	5.15	22.08	4.78	0.72 (−1.11, 2.54)	0.77 (163)	0.441
Duration of opioid exposure (years)	15.03	6.95	13.37	7.50	15.48	6.76	−2.11 (−4.71, 0.50)	−1.60 (163)	0.112
Duration of opioid addiction (years)	14.24	7.00	12.13	7.59	14.81	6.75	−2.68 (−5.29, −0.07)	−2.03 (163)	0.044
Duration of illicit drug use prior to joining MMT (years)	15.08	6.91	13.47	7.68	15.52	6.65	−2.04 (−4.63, 0.54)	−1.56 (163)	0.121
Duration in MMT (years)	2.92	2.09	2.43	1.54	3.05	2.21	−0.62 (−1.26, 0.03)	−1.91 (76)	0.060
Methadone dose (mg)	76.64	37.63	78.00	32.29	76.27	39.05	1.73 (−12.46, 15.92)	0.24 (163)	0.810
Averaged SOWS score^b^	5.43	6.91	4.26	8.65	5.74	6.35	−1.48 (−4.04, 1.08)	−1.14 (166)	0.256
Monthly income (RM)	844.42	474.71	968.57	501.48	811.00	463.57	157.57 (−19.81, 334.96)	1.75 (163)	0.081

**Notes.**

a *p* values were obtained using an unpaired independent *t*-test.

b The average of two measurements taken at 0 h (i.e., immediately (approximately 30 min) before taking their morning dose of methadone), and at 24 h after the dose intake.

*N* number of subject MMT methadone maintenance therapy SOWS subjective opiate withdrawal scale RM Ringgit Malaysia SD standard deviation CI confidence interval df degree of freedom

### Comparison of global PSQI scores between pain-sensitive and pain-tolerant participants

Table 2 shows the mean global PSQI scores, pain-tolerance time of the opioid-dependent participants at 0 h and at 24 h after the first CPT, and averaged pain-tolerance time (i.e., the average of two measurements taken at 0 h and at 24 h). Of 168 participants, three did not complete the PSQI questionnaire and therefore, their global PSQI scores were not included in the analysis. Our study participants had poor sleep quality with a mean global PSQI score of 5.47 (SD = 2.74) which was above the cut-off score of 5.

**Table 2:** The Pittsburgh Sleep Quality Index (PSQI) and pain-tolerance data in opioid-dependent participants.

				95% CI	
Variable	Mean	SD	SE	Lower limit	Upper limit
Global PSQI score (*N* = 165)	5.47	2.74	0.21	5.05	5.89
Pain-tolerance (second)					
At 0 h (*N* = 168)	36.09	37.11	2.86	30.44	41.74
At 24th hour (*N* = 163)	27.87	36.36	2.85	22.24	33.49
Averaged^a^ (*N* = 168)	32.16	35.25	2.72	26.79	37.53

**Notes.**

a The average of two measurements taken at 0 h and at 24 h after the first CPT.

*N* number of subject PSQI Pittsburgh Sleep Quality Index (PSQI) SD standard deviation SE standard error CI confidence interval

The pain-sensitive group reported poorer sleep quality, with a mean PSQI score (SD) of 5.78 (2.80), when compared to the pain-tolerant group with a mean score (SD) of 4.31 (2.18) (*p* = 0.005). With duration of opioid duration as the covariate, since this variable was significant between the two groups, ANCOVA revealed that the pain-sensitive group reported higher mean global PSQI scores (adjusted mean 5.76, 95% CI 5.29; 6.22) than the pain-tolerant group (adjusted mean 4.42, 95% CI 3.52; 5.32) (*p* = 0.010) (Table 3).

**Table 3:** Comparison of global PSQI scores between pain-sensitive and pain-tolerant participants with and without controlling for duration of opioid addiction.

A: Without controlling for duration of opioid addiction					
	Global PSQI				
Group	Mean	SD	Mean difference (95% CI)	*t*-statistic (df)	*p*-value^a^
Pain-tolerant (*N* = 35)	4.31	2.18	−1.47 (−2.48, −0.46)	−2.88 (163)	0.005
Pain-sensitive (*N* = 130)	5.78	2.80			

B: Controlling for duration of opioid addiction				
Group	Adj. mean^b^ (95% CI)	Adj. mean difference (95% CI)^c^	*F* stat. (df)	*p*-value^d^
Pain-tolerant (*N* = 35)	4.42 (3.52, 5.32)	−1.34 (−2.35, −0.32)	6.73 (1, 162)	0.010
Pain-sensitive (*N* = 130)	5.76 (5.29, 6.22)			

**Notes.**

a *p* values were obtained using an unpaired independent *t*-test.

b Adjusted mean controlling for duration of opioid addiction.

c Bonferroni adjustment for 95% confidence interval for difference.

d *p* values were obtained using an analysis of covariance.

*N* number of subject PSQI Pittsburgh Sleep Quality Index (PSQI) SD standard deviation CI confidence interval df degree of freedom

## Discussion

Sleep disorders are frequently reported by opioid-dependent patients during methadone treatment but it is unknown if pain-sensitivity plays a significant role. Sleep disturbances could have a profound impact on quality of life, health and even impair engagement with treatment programme leading to continued illicit drug use (Staedt et al., 1996; Hsu et al., 2012; Pud, Zlotnick & Lawental, 2012). Opioid therapy is not only associated with development of tolerance but also increased sensitivity to pain, a condition referred to as opioid-induced hyperalgesia (Compton, Charuvastra & Ling, 2001; Chu, Clark & Angst, 2006; Eyler, 2013). In the present study, we found, as hypothesized, that pain-sensitive male patients on methadone treatment had a poorer sleep quality compared to pain-tolerant patients.

The aetiology of sleep disorders among patients on methadone treatment is likely multifactorial and complex. Pain, co-morbid psychiatric symptoms, concomitant drug abuse and methadone dose were all reported to be associated with sleep disorders in this group of patients (Stein et al., 2004; Peles, Schreiber & Adelson, 2006; Peles, Schreiber & Adelson, 2009). More recently, the insomnia side-effect was found to be associated with genetic variations in the µ-opioid receptor (OPRM1) gene (Wang et al., 2012), which indicates that susceptible polymorphisms may also affect sleep control among patients on methadone. Our cross-sectional design did not allow us to determine whether methadone was responsible for changing the quality of sleep or that sleep quality could be the reason why patients adhered to methadone. However, previous studies found that alterations in sleep quality had existed even prior to MMT (Peles, Schreiber & Adelson, 2006; Peles, Schreiber & Adelson, 2009; Liao et al., 2011). It can be difficult to determine whether sleep disorders in our study population are due to a single cause or a combination of sleep disturbing factors, although it is more likely to be the latter. We have previously shown that age of first illicit drug use, age of first opioid abuse, duration of opioid exposure, duration of opioid addiction, duration in MMT and monthly income might be factors associated with a worse PSQI score (Zahari et al., in press). Unfortunately, PSQI, although a valid scale, did not allow us to address the many questions behind the poor-sleep that was experienced in our study population.

The strength of the present study is that it used the cold pressor test (CPT) as an objective measurement of pain-sensitivity. The CPT was considered a good choice since previous studies have demonstrated that with this test, all patients became hyperalgesic as well as tolerant one month after oral morphine therapy (Chu, Clark & Angst, 2006). Furthermore, previous studies only reported subjective pain experience but did not objectively measure pain (Stein et al., 2004; Peles, Schreiber & Adelson, 2006; Peles, Schreiber & Adelson, 2009). In many cases where opioid-dependent patients complaining about pain, it can be difficult to know who is faking a pain complaint (drug-seeking) to get an opioid prescription (Fields, 2011). Thus, it is possible that without any objective tests for pain-sensitivity, patients can fake their pain experience. The fact that a significant percentage of patients on MMT reported chronic pain is intriguing (Dunn, Brooner & Clark, 2014), and therefore an objective measure like CPT would be useful in addition to subjective pain questionnaire.

Our result showed that opioid-dependent males on methadone represent a more pain-sensitive subset of clinical patients. The present study did not find a significant relationship between pain-sensitivity and methadone dose. This finding is in agreement with the study by Compton and colleagues (2000). However, plasma methadone level was not measured which is a limitation. On the other hand, the duration of opioid addiction was found to be significantly longer in the pain-sensitive group which also reported poorer sleep quality. Previous works have shown an association between sleep disturbances and duration of substance use, including duration of opiate abuse (Peles, Schreiber & Adelson, 2006; Peles, Schreiber & Adelson, 2009) and duration of heroin abuse (Liao et al., 2011).

Previous studies have included patients with co-morbid psychiatric illnesses such as depression and anxiety (Stein et al., 2004; Peles, Schreiber & Adelson, 2006; Peles, Schreiber & Adelson, 2009). In our study, we carefully excluded patients with psychiatric illnesses, individuals who were taking benzodiazepines, cannabinoids and barbiturates, and individuals with chronic or ongoing acute pain, in order to minimize the possible bias of co-morbid illnesses had on sleep parameters in our analysis. It is a limitation that no specific scales were used to screen for psychiatric morbidities in our study. However, medical records were carefully checked and doctors contacted to verify the psychiatric and drug history provided by patients.

This study has other limitations. Our study did not use an objective sleep evaluation test such as polysomnography (PSG). However, a study reported that objective sleep measures concur with subjective sleep experience in MMT patients (Sharkey et al., 2011). Future studies should consider using PSG hand in hand with objective pain measure in addition to other sleep parameters such as sleep architecture. Only male subjects were included in the current study but this reflected the cohort population of drug abusers in Malaysia where more than 90% of them are males (Manan et al., 2013). Furthermore, this can minimise the possible effects of gender had on cold pressor pain response (Fillingim & Maixner, 1995; Fillingim et al., 2009; Alabas et al., 2012; Racine et al., 2012) and sleep (Krishnan & Collop, 2006).

Based on the results of our study, the PSQI scores should probably be evaluated in every opioid-dependent patient on methadone therapy complaining of pain that is confirmed using objective measure. When they indicate a poor sleep quality, we recommend referring these patients to a sleep laboratory in order to identify sleep problems through a PSG evaluation, if available, so that an intervention or a suitable treatment protocol can be established.

To conclude, pain-sensitivity is associated with impaired sleep quality in opioid-dependent males on methadone treatment. Sleep disturbance and pain-sensitivity in this population should not be disregarded.

## Supplemental Information

10.7717/peerj.839/supp-1 Supplemental Information 1 Raw data
